# The clinical value of neutrophil-to-lymphocyte ratio, platelet-to-lymphocyte ratio, and D-dimer-to-fibrinogen ratio for predicting pneumonia and poor outcomes in patients with acute intracerebral hemorrhage

**DOI:** 10.3389/fimmu.2022.1037255

**Published:** 2022-10-10

**Authors:** Sai Luo, Wen-Song Yang, Yi-Qing Shen, Ping Chen, Shu-Qiang Zhang, Zhen Jia, Qi Li, Jian-Ting Zhao, Peng Xie

**Affiliations:** ^1^ Department of Neurology, The Fourth Affiliated Hospital of Xinxiang Medical University, Xinxiang, China; ^2^ Department of Neurology, The First Affiliated Hospital of Chongqing Medical University, Chongqing, China; ^3^ Department of General Practice, The Fourth Affiliated Hospital of Xinxiang Medical University, Xinxiang, China; ^4^ Department of Radiology, Chongqing University FuLing Hospital, Chongqing, China; ^5^ Department of Radiology, The Fourth Affiliated Hospital of Xinxiang Medical University, Xinxiang, China

**Keywords:** intracerebral hemorrhage, neutrophil-to-lymphocyte ratio, platelet-to-lymphocyte ratio, D-dimer-to-fibrinogen ratio, pneumonia, poor outcome

## Abstract

**Background:**

This study aimed to investigate the neutrophil-to-lymphocyte ratio (NLR), platelet-to-lymphocyte ratio (PLR), and D-dimer-to-fibrinogen ratio (DFR) as predictors of pneumonia and poor outcomes in patients with acute intracerebral hemorrhage (ICH).

**Methods:**

We retrospectively examined patients with acute ICH treated in our institution from May 2018 to July 2020. Patient characteristics, laboratory testing data, radiologic imaging data, and 90-day outcomes were recorded and analyzed.

**Results:**

Among the 329 patients included for analysis, 183 (55.6%) developed pneumonia. Systolic blood pressure, initial hematoma volume, D-dimer concentration, NLR, PLR, DFR, and white blood cell, platelet, neutrophil, and lymphocyte counts at admission were significantly higher in patients who developed pneumonia than in those who did not; however, the Glasgow coma scale (GCS) score at admission was significantly lower in pneumonia patients compared with non-pneumonia patients (all P <0.05). Multivariate logistic regression showed that the NLR and PLR were independent predictors of pneumonia, and the NLR and DFR were independent predictors of poor 90-day outcomes (modified Rankin scale score 4–6).

**Conclusion:**

The NLR and PLR were independent predictors of pneumonia and the NLR and DFR were independent predictors of poor 90-day outcomes. The NLR, PLR, and DFR can provide prognostic information about acute ICH patients.

## Introduction

Intracerebral hemorrhage (ICH) accounts for 15% to 20% of all strokes (1) and is associated with high rates of mortality and disability (2). The reported mortality was 35% at 7 days and 59% at 1 year (3). Currently, there is no treatment for the reduction of ICH mortality (4).

Pneumonia is one of the most common consequences of acute stroke and can cause brain hypoxia and further brain damage. It, therefore, increases healthcare costs and the length of hospital stays (5–7) and poses a major threat to the patient’s health and life (8, 9). Furthermore, the incidence of pneumonia is higher after ICH than after cerebral infarction because ICH is characterized by sudden onset, rapid intracranial pressure increase, and rapid neurological deterioration (10). Several studies reported that neurological dysfunction and stroke severity in acute stroke patients are important predictors of post-stroke infection. However, the mechanisms underlying lung infection after ICH are not fully understood (11). A clinical study speculated that risk factors for poststroke pneumonia include aspiration, dysphagia, nasogastric tubing, and mechanical ventilation (12). However, conventional pneumonia prophylaxis and prophylactic antibiotics have not improved clinical outcomes in acute ICH patients (13, 14). Therefore, objective and conveniently accessible outcome predictors are needed.

The neutrophil-to-lymphocyte ratio (NLR) has the potential as such a predictor (15). A high NLR indicates an imbalance between central and peripheral inflammation and was shown to be an independent risk factor for pneumonia in acute stroke patients (16). Secondary brain injury is more likely to occur after infection and is associated with an increase in neutrophils and a decrease in lymphocytes.

The D-dimer-to-fibrinogen ratio (DFR) is another potential predictor. This novel coagulation parameter indicates the balance of fibrinolysis and coagulation processes (17) and has clinical value in patients with acute coronary syndrome and acute ischemic stroke (18). In addition, the platelet-to-lymphocyte ratio (PLR) may also have predictive value based on studies of patients with ischemic stroke and pulmonary embolism caused by intracardiac thrombotic disease (19, 20). PLR reflects platelet aggregation and systemic inflammation (21).

Previous studies have shown that the NLR, DFR, and PLR are associated with infection or poor outcomes in patients with the acute coronary syndrome, acute cerebral infarction, and pulmonary embolism. This study aimed to investigate the association of the NLR, DFR, and PLR with pneumonia and poor outcomes in patients with acute ICH.

## Patients and methods

### Study population

This retrospective study examined the medical records of 848 acute ICH patients hospitalized in intensive care units in the Fourth Clinical Faculty of Xinxiang Medical University from May 2018 to July 2020.

### Clinical data collection and radiological assessment

Data regarding comorbidities, cigarette and alcohol use, demographics, clinical features, laboratory testing, and radiologic imaging were recorded, including the time of ICH onset, Glasgow coma scale (GCS) score, length of hospital stay, and modified Rankin scale (mRS) score at 90-day follow-up. Baseline hematoma volume, presence of intraventricular hemorrhage (IVH) and/or subarachnoid hemorrhage (SAH), and in-hospital and 90-day mortality rates were recorded and evaluated as potential risk factors for pneumonia and predictors of poor outcomes. Hematoma volume was measured using the ABC/2 method (22). Blood counts and D-dimer and fibrinogen concentrations were determined using a fully automated biochemical analyzer according to the manufacturer’s instructions (AU2700, Olympus, Tokyo, Japan).

### Patient selection

**Figure 1:**
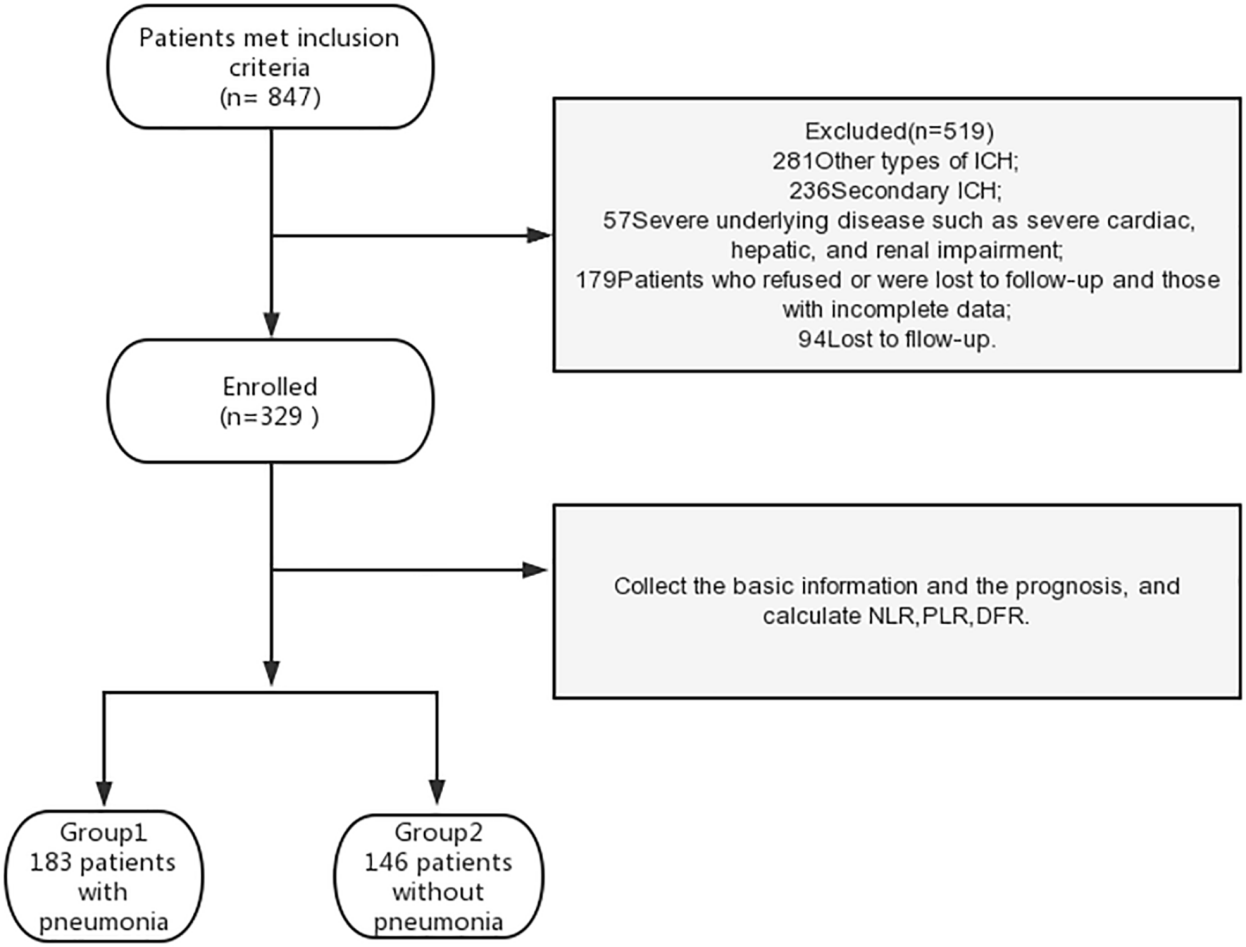
The flow chart of excluded patients.

Inclusion criteria were as follows: 1) diagnosis of acute hemorrhagic stroke according to the Chinese Guidelines for the Diagnosis and Treatment of ICH (23), 2) deep hemorrhage in the basal ganglia and/or thalamus and/or infratentorial confirmed by cranial computed tomography within 12 hours of admission, and 3) available chest computed tomography imaging data. We excluded patients with other types of ICH (traumatic, arteriovenous malformation, cerebral aneurysm, tumor, primary IVH, cavernous hemangioma, hemorrhagic cerebral infarction, and lobar hemorrhage) and those with severe underlying diseases such as severe cardiac, hepatic, and renal impairment. Patients who refused or were lost to follow-up and those with incomplete data were also excluded (**Figure 1**). The study was conducted in accordance with the 1964 Helsinki Declaration. Medical ethics committee approval was obtained. All patients provided written informed consent.

### Study grouping

Among the 848 eligible patients, 519 were excluded based on the exclusion criteria. Therefore, 329 patients were included in the analysis (**Figure 1**). Patients were grouped according to the presence of pneumonia (pneumonia or no pneumonia groups; **Figure 1**). Diagnostic criteria for nosocomial pneumonia (24) included chest X-ray or computed tomography (CT) showing new or progressive patchy infiltrates, solid lobar changes, or pleural effusion, plus two or more of the following three clinical symptoms, to establish a clinical diagnosis: 1. fever, body temperature >38°C; 2. purulent airway secretions; and 3. low leukocyte count (<4000 × 10^9^/L), high leukocyte count (>10× 10^9^/L). Patients were also grouped into poor and good outcome groups based on their mRS score at 90 days. A poor outcome was defined as an mRS score of 4–6 and a good outcome was defined as an mRS score of 0–3 (25).

### Statistical analysis

Statistical analyses were performed using SPSS software version 25 (IBM Corp. Armonk, NY, USA). Continuous variables are expressed as means with standard deviation and were compared using a nonparametric rank-sum test and independent sample *t*-test as appropriate. Categorical variables are expressed as numbers with percentages and were compared using the chi-square test or Fisher’s exact test as appropriate. Risk factors and predictors were determined using multivariate logistic regression to calculate the odds ratio (OR) with 95% confidence interval (CI). P <0.05 was considered statistically significant.

## Results

**Figure 2:**
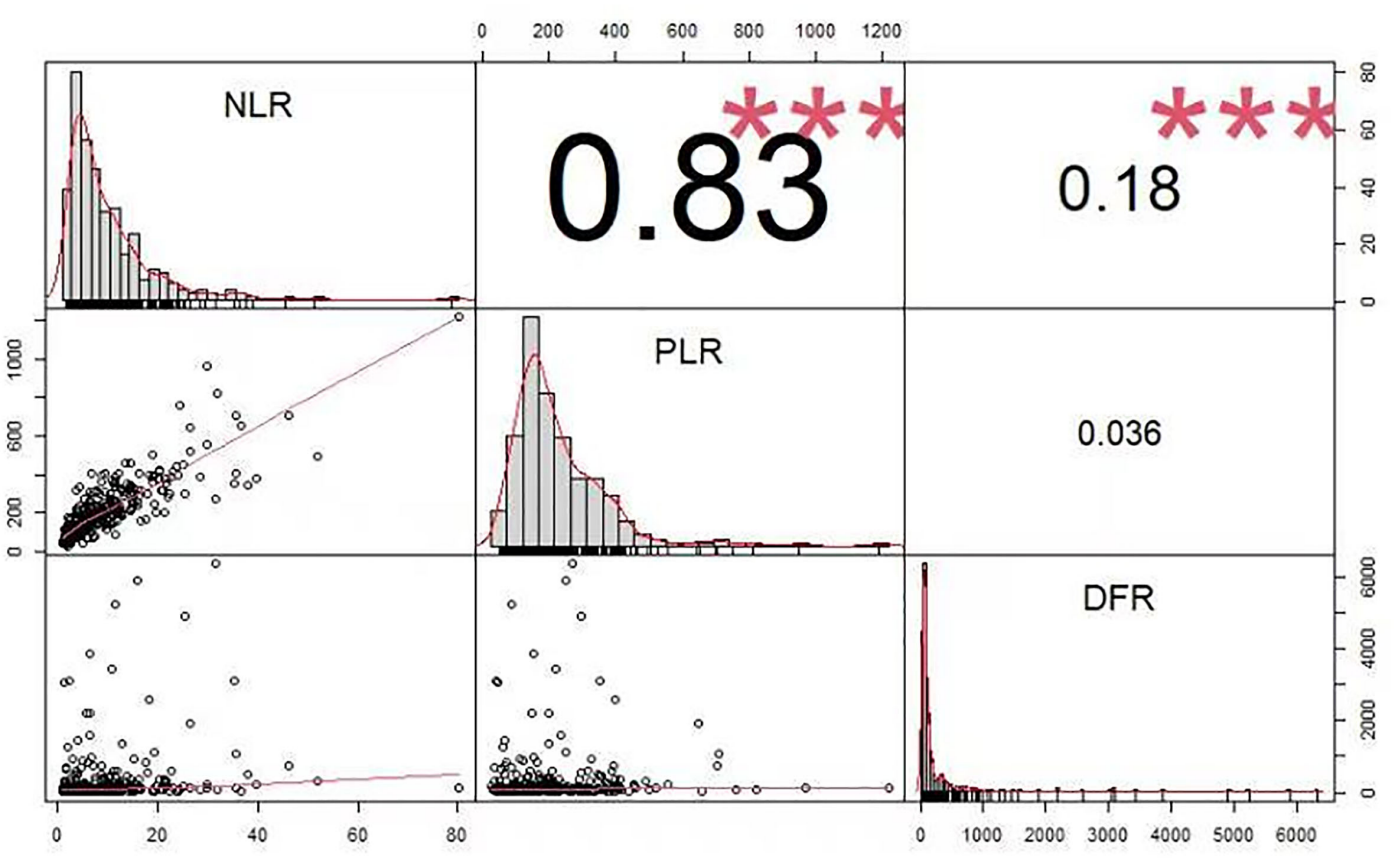
The correlation coefficient between The PLR, NLR, and DFR.

**Figure 2** shows the correlations between the PLR, NLR, and DFR. NLR was significantly correlated with PLR, the NLR was significantly correlated with DFR, and there was no significant correlation between the PLR and DFR. The correlation coefficients were as follows: NLR and PLR = 0.83, NLR and DFR = 0.18, PLR and DFR = 0.036 (**Figure 2**).

**Figure 3:**
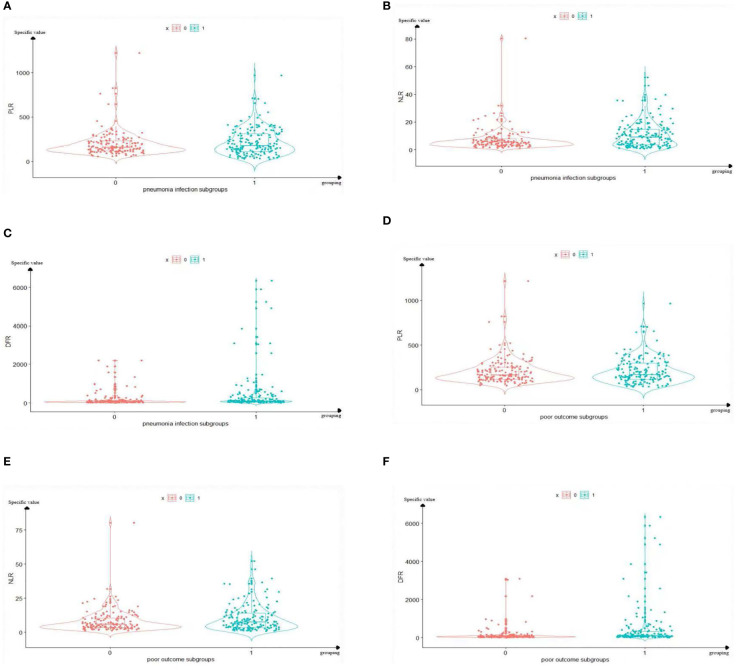
Boxplots of The PLR, NLR, and DFR in pulmonary infection group and prognosis group. The mean value and distribution of PLR, NLR and DFR of 329 sample sizes in each grouping are reflected. In the **(A–C)**, Group 1 represents the pneumonia group, and group 0 represents the non-pneumonia group. **(A)** The mean PLR of Group 1 was higher than that of group 0. **(B)** The mean NLR of Group 1 was higher than that of group 0. **(C)** The mean DFR of Group 1 was higher than that of group 0. In the panels **(D–F)**, Group 1 represents the poor outcome, and group 0 represents the good outcome. **(D)** The mean PLR of Group 1 was higher than that of group 0. **(E)** The mean PLR of Group 1 was higher than that of group 0. **(F)** The mean PLR of Group 1 was higher than that of group 0. PLR, platelet-to-lymphocyte ratio; NLR, neutrophil-to-lymphocyte ratio; DFR, D-dimer-to-fibrinogen ratio.

The distribution characteristics of the PLR, NLR, and DFR are shown in **Figure 3**. The mean value of group 1 (pneumonia group) was higher than that of group 0 (non-pneumonia group). Data of the PLR, NLR, and DFR in group 1 (pneumonia group) were relatively discrete, whereas data in group 0 (non-pneumonia group) were relatively centralized (**Figure 3**). The mean value of group 1 (poor outcome group) was higher than that of group 0 (good outcome group). Data of the PLR, NLR, and DFR in group 1 (poor outcome group) were relatively discrete, whereas data in group 0 (good outcome group) were relatively centralized (**Figure 3**).

Compared with patients in the non-pneumonia group, those in the pneumonia group had a significantly larger initial hematoma volume (P <0.001) and lower GCS score at admission (P <0.001), with significantly higher baseline platelet (P =0.025), leukocyte (P <0.001), neutrophil (P <0.001), and lymphocyte (P <0.001) counts. In addition, the D-dimer concentration (P <0.001), NLR (P <0.001), PLR (P =0.008), DFR (P <0.001), and systolic blood pressure (P =0.015) were significantly higher in the pneumonia group. The incidences of IVH (P <0.001), SAH (P <0.001), hypertension (P =0.036), and poor outcomes (P <0.001) in the pneumonia group were significantly higher than those in the non-pneumonia group. The in-hospital mortality (P =0.003) and 90-day mortality (P <0.001) rates were also significantly higher in the pneumonia group than in the non-pneumonia group. The number of patients with basal ganglia hemorrhage in the non-pneumonia group was 1.5 times higher than that in the pneumonia group (**Table 1**).

**Table 1:** Comparison of demographic, clinical and imaging characteristics and outcome between patients with and without pneumonia.

Demographic	Pneumonia (n = 183 55.6%)	Non-pneumonia (n = 146, 44.4%)	t/x^2^/z	p Value
**Demographic**				
Age, year, Mean(SD)	60.54 ± 13.49	61.52 ± 11.40	-0.696	**0.023**
Sex, male, n(%)	121 (66.1%)	89 (61.0%)	0.937	0.333
**Clinical characteristics**				
Alcohol consumption, n (%)	13 (7.1%)	14 (9.6%)	0.415	0.666
Smoking, n (%)	22 (12.0%)	18 (12.3%)	0.007	0.933
Diabetes mellitus, n (%)	21 (11.5%)	22 (15.1%)	0.923	0.337
History of hypertension, n(%)	110 (60.1%)	104 (71.2%)	4.420	**0.036**
Admission SBP, mmHg, Mean(SD)	172.56 ± 28.36	164.18 ± 24.27	2.498	**0.015**
Admission DBP, mmHg, Mean(SD)	96.66 ± 16.88	96.64 ± 16.26	0.012	0.255
Admission GCS score, median (IQR)	6 [4-10]	14 [10.75-15]	101.432	**<0.001**
Platelets,10^9^/l, Mean(SD)	216.10 ± 86.33	209.60 ± 57.56	0.782	**0.025**
Leukocytes, 10^9^/L, Mean(SD)	11.68 ± 5.12	8.87 ± 3.09	5.846	**<0.001**
Neutrophils, 10^9^/L, Mean(SD)	9.66 ± 5.04	7.06 ± 3.06	5.481	**<0.001**
Lymphocyte, 10^9^/L, Mean(SD)	1.50 ± 1.29	1.32 ± 0.61	1.596	**<0.001**
D-Dimer, ng/mL, Mean(SD)	1017.36 ± 2238.75	453.03 ± 858.56	2.881	**<0.001**
Fibrinogen, g/L, Mean(SD)	3.00 ± 1.42	2.78 ± 0.63	1.790	0.085
NLR, Mean(SD)	10.91 ± 9.43	7.19 ± 7.94	3.80	**<0.001**
PLR, Mean(SD)	218.53 ± 147.53	197.16 ± 141.10	1.331	**0.008**
DFR, Mean(SD)	401.86 ± 980.25	176.68 ± 358.74	2.638	**<0.001**
**Imaging features**				
Baseline ICH volume, mL, median (IQR)	32.69 [12.26-63.31]	10.21 [4.17-20.21]	62.832	**<0.001**
IVH presence, n (%)	132 (72.1%)	51 (34.9%)	45.527	**<0.001**
SAH presence, n (%)	53 (29.0%)	16 (11.0%)	15.880	**<0.001**
**ICH Locations**				
Basal ganglia hemorrhage, n (%)	44 (24.0%)	66 (45.2%)	16.341	**<0.001**
Thalamic hemorrhage, n (%)	28 (15.3%)	29 (19.9%)	1.180	0.277
Lobar hemorrhage, n (%)	8 (4.4%)	12 (8.2%)	2.106	0.147
Infratentorial hemorrhage, n (%)	20 (10.9%)	15 (10.3%)	0.037	0.848
**Outcome**				
In-hospital mortality, n (%)	16 (8.7%)	2 (1.4%)	8.537	**0.003**
90-day mortality, n (%)	73 (39.9%)	14 (9.6%)	38.335	**<0.001**
90-day poor outcome, n (%)	134 (73.2%)	41 (28.1%)	66.466	**<0.001**
90-day mRS score, median (IQR)	5 [3-6]	2 [1-4]	74.325	**<0.001**

ICH, intracerebral hemorrhage; CT, computed tomography; GCS, Glasgow Coma Scale; IVH, intraventricular hemorrhage; SAH, subarachnoid hemorrhage; INR, interquartile range; SD, standard deviation; mRS, modified Rankin scale; SBP, systolic blood pressure; DBP, diastolic blood pressure; NLR, neutrophil-to-lymphocyte ratio; PLR, platelet-to-lymphocyte ratio; DFR, D-dimer-to-fibrinogen ratio The bold values indicated was considered statistically significant.

The NLR (OR 1.076, 95% CI, 1.003–1.154, P =0.041), PLR (OR 0.996, 95% CI, 0.992–0.999, P =0.023), GCS score at admission (OR 0.782, 95% CI, 0.726–0.841, P <0.001), and initial hematoma volume (OR 1.035, 95% CI, 1.020–1.049, P <0.001) were independent risk factors for pneumonia (**Table 2**). There was a significant correlation between NLR and PLR (**Figure 2**).

**Table 2:** Multivariable logistic regression models of primary ICH for predicting pneumonia.

Variable	Odds Ratio	95% Confidence Interval [25%, 75%]	p-Value
NLR	1.076	[1.003, 1.154]	**0.041**
PLR	0.996	[0.992, 0.999]	**0.023**
DFR	1.000	[0.999, 1.000]	0.859
Age, year	0.998	[0.964, 1.013]	0.337
History of hypertension	0.617	[0.333, 1.146]	0.126
Admission SBP, mmHg	1.009	[0.998, 1.020]	0.106
Admission GCS score	0.782	[0.726, 0.841]	**<0.001**
Baseline ICH volume, mL	1.035	[1.020, 1.049]	**<0.001**
Basal ganglia hemorrhage	0.584	[0.319, 1.070]	0.082

ICH, intracerebral hemorrhage; SBP, systolic blood pressure; GCS, Glasgow Coma Scale; NLR, neutrophil-to-lymphocyte ratio; PLR, platelet-to-lymphocyte ratio; DFR, D-dimer-to-fibrinogen ratio The bold values indicated was considered statistically significant.

**Table 3** shows the characteristics of patients grouped according to outcome. Compared with patients with a good outcome, those with a poor outcome were significantly older (P =0.024), with significantly larger initial hematoma volume (P <0.001) and lower GCS score at admission (P <0.001), with significantly higher baseline platelet (P =0.024), leukocyte (P <0.001), neutrophil (P <0.001), and lymphocyte (P <0.001) counts. The D-dimer concentration (P <0.001) and fibrinogen levels (P =0.027) were significantly higher in the poor outcome group. In addition, the NLR (P =0.012) was significantly higher in the poor outcome group than in the good outcome group. The DFR (P <0.001) was significantly lower in the poor outcome group than in the good outcome group. The proportions of patients with IVH (P <0.001) and SAH (P <0.001) were significantly higher in the poor outcome group than in the good outcome group. Compared with the poor outcome group, patients with basal ganglia hemorrhage were almost two-fold as likely to have a good outcome.

**Table 3:** Comparison of demographic, clinical, and imaging characteristics and outcomes between patients with and without poor outcome.

Demographic	Poor outcome (n = 175,53.2%)	Good outcome (n =, 154,46.8%)	t/x^2^/z	p Value
**Demographic**				
Age, year, Mean(SD)	63.38 ± 13.38	58.24 ± 11.06	3.762	**0.024**
Sex, male, n(%)	113 (64.6%)	97 (63%)	0.089	0.765
**Clinical characteristics**				
Alcohol consumption, n (%)	11 (6.3%)	16 (10.4%)	1.831	0.176
Smoking, n (%)	19 (12.0%)	21 (12.3%)	0.009	0.925
Diabetes mellitus, n (%)	27 (10.4%)	16 (15.4%)	1.831	0.176
History of hypertension, n(%)	110 (62.9%)	104 (67.5%)	0.788	0.375
Admission SBP, mmHg, Mean(SD)	173.64 ± 27.01	164.35 ± 25.85	3.175	0.213
Admission DBP, mmHg, Mean(SD)	97.34 ± 17.84	95.87 ± 15.04	0.806	0.489
Admission GCS score, median (IQR)	6[4-11]	13[8-15]	58.186	**<0.001**
Platelets,10^9^/L, Mean(SD)	216.32 ± 86.90	209.68 ± 58.45	0.802	**0.024**
Leukocytes, 10^9^/L, Mean(SD)	11.35 ± 5.20	9.40 ± 3.42	3.954	**<0.001**
Neutrophils, 10^9^/L, Mean(SD)	9.33 ± 5.10	7.57 ± 3.39	3.611	**<0.001**
Lymphocyte, 10^9^/L, Mean(SD)	1.49 ± 1.18	1.34 ± 0.86	1.346	**0.003**
D-Dimer, ng/mL, Mean(SD)	1096.93 ± 2238.20	391.92 ± 930.81	3.641	**<0.001**
Fibrinogen, g/L, Mean(SD)	3.07 ± 1.43	2.71 ± 0.61	2.918	**0.027**
NLR, Mean(SD)	10.26 ± 9.47	8.12 ± 8.28	2.165	**0.012**
PLR, Mean(SD)	212.83 ± 146.39	204.75 ± 143.51	0.504	0.115
DFR, Mean(SD)	160.55 ± 404.56	426.36 ± 979.18	3.141	**<0.001**
**Imaging features**				
Baseline ICH volume, mL, median (IQR)	30.57 [11.44-62.31]	10.59 [3.92-23.50]	7.544	**<0.001**
IVH presence, n (%)	123 (70.3%)	60 (39%)	32.563	**<0.001**
SAH presence, n (%)	56 (32%)	13 (8.4%)	27.430	**<0.001**
**ICH Locations**				
Basal ganglia hemorrhage, n (%)	39 (22.3%)	71 (46.1%)	20.88	**<0.001**
Thalamic hemorrhage, n (%)	29 (16.6%)	28 (18.2%)	0.148	0.700
Lobar hemorrhage, n (%)	9 (5.1%)	11 (7.1%)	0.574	0.449
Infratentorial hemorrhage, n (%)	13 (8.4%)	22 (12.6%)	1.470	0.225

ICH, intracerebral hemorrhage; CT, computed tomography; GCS, Glasgow Coma Scale; IVH, intraventricular hemorrhage; SAH, subarachnoid hemorrhage; IQR, interquartile range; SD, standard deviation; mRS, modified Rankin scale; SBP, systolic blood pressure; DBP, diastolic blood pressure; NLR, neutrophil-to-lymphocyte ratio; PLR, platelet-to-lymphocyte ratio; DFR, D-dimer-to-fibrinogen ratio The bold values indicated was considered statistically significant.

The NLR (OR 1.079, 95% CI, 1.021–1.140, P =0.007), DFR (OR 1.003, 95% CI, 1.001–1.005, P =0.016), age (OR 0.967, 95% CI, 0.944–0.990, P =0.006), and initial hematoma volume (OR 0.983, 95% CI, 0.969–0.996, P =0.011) were independent predictors of poor outcomes (**Table 4**). There may be a significant correlation between the NLR and DFR (**Figure 2**).

**Table 4:** Multivariable logistic regression models of spontaneous ICH for predicting poor outcome.

Variable	Odds Ratio	95% Confidence Interval [25%, 75%]	p-Value
NLR	1.079	[1.021, 1.140]	**0.007**
DFR	1.003	[1.001, 1.005]	**0.016**
Admission GCS score	1.096	[0.989, 1.214]	0.081
Age, year	0.967	[0.944, 0.990]	**0.006**
Baseline ICH volume, mL	0.983	[0.969, 0.996]	**0.011**
SAH presence	0.566	[0.245, 1.308]	0.183
IVH presence	0.781	[0.375, 1.627]	0.509
Basal ganglia hemorrhage	1.366	[0.737, 2.532]	0.321
NLR*DFR	1.000	[0.999, 1.000]	0.008

ICH, intracerebral hemorrhage; NLR, neutrophil-to-lymphocyte ratio; DFR, D-dimer-to-fibrinogen ratio; GCS, Glasgow Coma Scale; IVH, intraventricular hemorrhage; SAH, subarachnoid hemorrhage The bold values indicated was considered statistically significant.

## Discussion

Our study found that age, initial hematoma volume, D-dimer concentration, systolic blood pressure, GCS score at admission, NLR, PLR, DFR, and platelet, leucocyte, neutrophil, and lymphocyte counts were significantly associated with pneumonia in patients with acute ICH. The prevalence of hypertension, IVH presence, and SAH presence was significantly higher in the pneumonia group than in the non-pneumonia group. There were slightly fewer patients in the pneumonia group with bleeding in the basal ganglia than in the non-pneumonia group. Of the significantly higher factors identified, the NLR, PLR, GCS score, and initial hematoma volume were independent risk factors for pneumonia in the multivariate analysis, and the NLR, DFR, age, and initial hematoma volume were independent predictors of a poor outcome.

Pneumonia is the most prevalent complication of early stroke and is associated with high mortality, increased cost of treatment, and poor functional outcomes (26, 27). In patients with acute ICH, immunity is impaired by the stress response and elevated blood glucose levels, which promote bacterial growth and proliferation that exacerbate pulmonary microcirculatory dysfunction and increase the risk of pneumonia (28). Zhang et al. (29) observed changes in the immune system and intestinal barrier function during the development of ICH in mice. They found that ICH mice had rapidly dysregulated proinflammatory and immunosuppressive responses, which changed the structural distribution of lung microbiota and intestinal microbiota, promoted bacterial migration, and increased their susceptibility to respiratory diseases. ICH involves multiple pathophysiological pathways. Neutrophils, macrophages, monocytes, and microglia are activated, accumulate around the hematoma, and induce inflammation, which has an impact on the degree of neurological damage (30, 31). Our study examined whether systemic parameters of inflammation could be used to predict pneumonia in ICH patients.

The NLR was used as a predictor of different inflammatory diseases in a previous study (15) and it was a predictor of death and intensive care unit admission in patients with coronavirus disease 2019 (32). Moreover, NLR might be a good indicator of COVID-19 disease severity (33). Zhang et al. (34) demonstrated that an association between the NLR and acute stroke-associated pneumonia did not disappear in patients with a large artery atherosclerosis infarct. Although inflammation is dynamically altered in the early stages of acute stroke, the results of Wang et al. (30) suggest that early NLR may be an independent risk factor for acute pneumonia during this period. The NLR reflects the balance between innate and adaptive immune responses, which is partially consistent with our findings.

In addition to an inflammatory state, ICH patients are in a hypercoagulable state associated with the release of coagulation factors. A large network of relationships exists between inflammation and thrombosis; therefore, the inflammatory response can promote a hypercoagulable state and subsequent thrombosis (35). Likewise, thrombosis factors can trigger an inflammatory response, which leads to a vicious cycle of inflammation and thrombosis (36, 37). The PLR reflects the coagulation and inflammatory pathways (38) and was shown to be an independent risk factor for hyperinflammatory processes (39). Moreover, it has a significant impact on thrombophilia, but is not influenced by other interfering factors (40). Therefore, the PLR provides a more accurate assessment of the inflammatory response and thrombotic status (41). Tao et al. (42) analyzed preoperative blood tests in 247 patients with aneurysmal subarachnoid hemorrhage and found that higher PLR was associated with neurological damage within 90 days of hemorrhage onset. In a case-control study of 335 acute ICH patients, Zou et al. (43) reported that those with higher NLR and PLR had a higher risk of gastrointestinal bleeding. Furthermore, higher NLR and PLR were associated with worse overall survival and outcomes, findings that are generally consistent with our findings.

The D-dimer concentration reflects the activation of hemostasis and fibrinolysis and is typically elevated in patients undergoing active thrombosis, such as stroke or venous thromboembolism. However, it may also be elevated in patients with other diseases, such as cancer or severe infection (44). The D-dimer concentration can be used to monitor disease progression and antifibrinolytic effects. Fibrinogen is a clotting factor produced by the liver that is converted to fibrin by activated thrombin. This conversion is the most critical step in the coagulation cascade. Fibrinolytic enzymes dissolve cross-linked fibrin to produce degradation products, such as D-dimer. As blood coagulates, fibrinogen is depleted and its blood concentration decreases. Fibrin stimulates platelet aggregation and damages endothelial cells, which promotes intravascular thrombosis and plaque development, processes that underly acute ischemic stroke. These abilities make fibrinogen useful for treating patients with severe stroke (44). Moreover, the fibrinogen concentration appears to be a predictor of ischemic stroke risk and has been associated with poor outcomes and death after stroke (45–47). Chen et al. (18) studied 155 patients with AIS and 33 patients with other disorders, and divided them into disorders of consciousness or unconsciousness. A comparison between groups with differences in plasma D-dimer, FIB, and DFR, showed that for different types of patients with disturbances of consciousness, D-dimer and fibrinogen alone were of limited value for a diagnosis of AIS, DFR was more specific for thrombosis than D-dimer plasma levels, and that the combination of D-dimer and DFR may improve the diagnostic efficiency and provide more etiological information. This would provide a new direct and cost-effective method for diagnosing thrombophilia in clinical practice.

In 2021, Wen et al. (48) used the predictive value of the DFR for hemorrhagic stroke and its related thrombotic complications. Their study showed a significant association between the DFR and lower limb deep vein thrombosis in young ICH patients. However, the relationship between the DFR and inflammatory complications in ICH patients has not been studied to date.

Our findings suggest that the NLR and PLR are independent risk factors for pneumonia and that the NLR and DFR are independent predictors of poor outcomes in patients with acute ICH. These ratios may have a role in real-world clinical practice and should be examined further in future studies. We also reported various clinical and imaging characteristics associated with pneumonia and outcomes in ICH patients, which may assist clinicians in identifying patients who require early and aggressive treatment.

Our study had several limitations. First, its retrospective single-center design and small sample size may have introduced confounding bias. Second, data were derived from medical records; therefore, pre-stroke medications could not be entirely accounted for and medications may have affected the laboratory results. Future large-scale prospective studies are warranted.

## Conclusion

The NLR and PLR are independent risk factors for pneumonia and the NLR and DFR are independent predictors of poor 90-day outcomes in patients with acute ICH.

## Data availability statement

The raw data supporting the conclusions of this article will be made available by the authors, without undue reservation.

## Ethics statement

The studies involving human participants were reviewed and approved by The Fourth Affiliated Hospital of Xinxiang Medical University ethics committee. The patients/participants provided their written informed consent to participate in this study.

## Author contributions

W-SY, JZ, and PX, were responsible for the study concept and design and had full access to all of the data in the study. SL, W-SY, Y-QS, PC, S-QZ, ZJ, QL, JZ, and PX performed the acquisition, analysis, or interpretation of data. SL drafted the manuscript. W-SY, JZ, and PX critically revised the manuscript. W-SY, SL, and Y-QS performed the statistical analyses. JZ, and PX were responsible for the administrative, technical, or material support.

## Acknowledgments

We appreciate the help and support from all participants who took part in the study. We thank J. Ludovic Croxford, PhD, from Edanz (www.edanz.com/ac) for editing a draft of this manuscript.

## Conflict of interest

The authors declare that the research was conducted in the absence of any commercial or financial relationships that could be construed as a potential conflict of interest.

## Publisher’s note

All claims expressed in this article are solely those of the authors and do not necessarily represent those of their affiliated organizations, or those of the publisher, the editors and the reviewers. Any product that may be evaluated in this article, or claim that may be made by its manufacturer, is not guaranteed or endorsed by the publisher.

## References

[1] Caplan LR. Intracerebral haemorrhage. Lancet (1992) 339:656–8. doi: 10.1016/0140-6736(92)90804-C 1347346

[2] Qureshi AI Mendelow AD Hanley DF. Intracerebral haemorrhage. Lancet (2009) 373:1632–44. doi: 10.1016/S0140-6736(09)60371-8 PMC3138486 19427958

[3] de Oliveira Manoel AL Goffi A Zampieri FG Turkel-Parrella D Duggal A Marotta TR. The critical care management of spontaneous intracranial hemorrhage: A contemporary review. Crit Care (2016) 20:272. doi: 10.1186/s13054-016-1432-0 27640182 PMC5027096

[4] Santoni M Andrikou K Sotte V Bittoni A Lanese A Pellei C. Toll li ke receptors and pancreatic diseases: From a pathogenetic mechanism to a therapeutic target. Cancer Treat Rev (2015) 41:569–76. doi: 10.1016/j.ctrv.2015.04.004 26036357

[5] Teh WH Smith CJ Barlas RS Wood AD Bettencourt-Silva JH Clark AB. Impact of stroke-associated pneumonia on mortality, length of hospitalization, and functional outcome. Acta Neurol Scand (2018) 138:293–300. doi: 10.1111/ane.12956 29749062

[6] Wilson RD. Mortality and cost of pneumonia after stroke for different risk groups. J Stroke Cerebrovasc Dis (2012) 21:61–7. doi: 10.1016/j.jstrokecerebrovasdis.2010.05.002 PMC3255072 22225864

[7] Bray BD Smith CJ Cloud GC Enderby P James M Paley L. The association between delays in screening for and assessing dysphagia after acute stroke, and therisk of stroke-associated pneumonia. J Neurol Neurosurg Psychiatry (2017) 88:25–30. doi: 10.1136/jnnp-2016-313356 27298147

[8] Tang Y Yin F Fu D Gao X Lv Z Li X. Efficacy and safety of minimal invasive surgerytreatment in hypertensive intracerebral hemorrhage: A systematic reviewand meta-analysis. BMC Neurol (2018) 18:136. doi: 10.1186/s12883-018-1138-9 30176811 PMC6120062

[9] Wang W Zhou N Wang C. Minimally invasive surgery for patients withhypertensive intracerebral hemorrhage with large hematoma volume: A retrospective study. World Neurosurg (2017) 105:348–58. doi: 10.1016/j.wneu.2017.05.158 28602881

[10] Morotti A Phuah CL Anderson CD Jessel MJ Schwab K Ayres AM. Leukocyte count and intracerebral hemorrhage expansion. Stroke (2016) 47:1473–8. doi: 10.1161/STROKEAHA.116.013176 PMC4879062 27103016

[11] Ionita CC Siddiqui AH Levy EI Hopkins LN Snyder KV Gibbons KJ. AcuteIschemic stroke and infections. J Stroke Cerebrovasc Dis (2011) 20:1–9. doi: 10.1016/j.jstrokecerebrovasdis.2009.09.011 20538486

[12] Tsai AS Berry K Beneyto MM Gaudilliere D Ganio EA Culos A. AYear-long immune profile of the system icRe sponse in acute stroke survivors. Brain (2019) 142:978–91. doi: 10.1093/brain/awz022 PMC6933508 30860258

[13] Kalra L Irshad S Hodsoll J Simpson M Gulliford M Smithard D. STROKE-INF investigators (2015) prophylactic antibiotics after acute stroke for reducing pneumonia in patients with dysphagia (STROKE-INF): A prospective, cluster-randomised, open-label, masked endpoint, controlled clinical trial. Lancet (2015) 386(10006):1835–44. doi: 10.1016/S0140-6736(15)00126-9 26343840

[14] Westendorp WF Vermeij JD Zock E Hooijenga IJ Kruyt ND Bosboom HJ. Thepreventive antibiotics in stroke study (PASS): A pragmatic randomised open-labelmasked endpoint clinical trial. Lancet (2015) 385:1519–26. doi: 10.1016/S0140-6736(14)62456-9 25612858

[15] Curbelo J Luquero Bueno S Galván-Román JM Ortega-Gómez M Rajas O Fernández-Jiménez G. Inflammation biomarkers in blood as mortality predictors in community-acquired pneumonia admitted patients: importance of comparison with neutrophil count percentage or neutrophil-lymphocyte ratio. PloS One (2017) 12:e0173947. doi: 10.1371/journal.pone.0173947 28301543 PMC5354424

[16] Lattanzi S Brigo F Trinka E Cagnetti C Di Napoli M Silvestrini M. Neutrophil-to-Lymphocyte ratio in acute cerebral hemorrhage: A system review. Transl Stroke Res (2019) 10(2):137–45. doi: 10.1007/s12975-018-0649-4 30090954

[17] Rao JH Ma YS Long J Liu JN Xue YZ Guo ZG. The clinical value of d-dimer/fibrinogen ratio and hypersensitive c-reactive protein in patients with acute coronary syndromes. Med JChin PLA (2018) 43(11):943–9.

[18] Chen X Li S Chen W Xu F Wang Y Zou G. The potential value of d-dimer to fibrinogen ratio in diagnosis of acute ischemic stroke. J Stroke Cerebrovasc Dis (2020) 29(8):104918. doi: 10.1016/j.jstrokecerebrovasdis.2020.104918 32430237

[19] Wuillemin WA Korte W Waser G Lämmle B. Usefulness of the d-dimer/fibrinogen ratio to predict deep venous thrombosis. J Thromb Haemost (2005) 3(2):385–7. doi: 10.1111/j.1538-7836.2004.01121.x 15670051

[20] Alvarez-Perez F Castelo-Branco M Alvarez-Sabín J. Usefulness of measurement of fibrinogen, d-dimer, d-dimer/fibrinogen ratio, c reactive protein and erythrocyte sedimentationrate to assess the pathophysiology and mechanism of ischaemic stroke. J Neurol Neurosurg Psychiatry (2011) 82:986–92. doi: 10.1136/jnnp.2010.230870 21296900

[21] Altintas Kadirhan O Altıntaş M Tasal A Kucukdagli O Asil T. The relationship of platelet-to-lymphocyte ratio with clinical outcome and final infarct core in acute ischemic stroke patients who have undergone endovascular therapy. Neurol Res (2016) 38:759–65. doi: 10.1080/01616412.2016.1215030 27477691

[22] Kothari RU Brott T Broderick JP Barsan WG Sauerbeck LR Zuccarello M. The ABCs of measuring intracerebral hemorrhage volumes. Stroke (1996) 27:1304–5. doi: 10.1161/01.STR.27.8.1304 8711791

[23] Chinese Society of Neurology, Chinese Stroke Society. Chinese Guidelines for diagnosis and treatment of acute intracerebral hemorrhage. Chin J Neurol (2019) 52:994–1005.

[24] Smith CJ Kishore AK Vail A Chamorro A Garau J Hopkins SJ. Diagnosis ofstroke-associated pneumonia: Recommendations from the pneumonia in stroke consensus group. Stroke (2015) 46:2335–40. doi: 10.1161/STROKEAHA.115.009617 26111886

[25] Qureshi AI Palesch YY Barsan WG Hanley DF Hsu CY Martin RL. ATACH−2 trial investigators and the neurological emergency treatment trials network. Intensive blood−pressure lowering in patients with acute cerebral hemorrhage. N Engl J Med (2016) 375:1033–43. doi: 10.1056/NEJMoa1603460 PMC5345109 27276234

[26] Boulouis G Morotti A Brouwers HB Charidimou A Jessel MJ Auriel E. Noncontrast computed tomography hypodensities predict poor outcome in intracerebral hemorrhage patients. Stroke (2016) 47:2511–6. doi: 10.1161/STROKEAHA.116.014425 PMC5039101 27601380

[27] Hannawi Y Hannawi B Rao CP Suarez JI Bershad EM. Stroke associated pneumonia:major advances and obstacles. Cerebrovasc Dis (2013) 35:430–43. doi: 10.1159/000350199 23735757

[28] Koennecke HC Belz W Berfelde D Endres M Fitzek S Hamilton F. Factors influencing in-hospital mortality and morbidity in patients treated on a stroke unit. Neurology (2011) 77:965–72. doi: 10.1212/WNL.0b013e31822dc795 21865573

[29] Zhang H Huang Y Li X Han X Hu J Wang B. Dynamic process of secondary pulmonary infection in mice with intracerebral hemorrhage. Front Immunol (2021) 12:767155. doi: 10.3389/fimmu.2021.767155 34868020 PMC8639885

[30] Wang Q Liu Y Han L He F Cai N Zhang Q. Risk factors for acute stroke-associated pneumonia and prediction of neutrophil-to-lymphocyte ratios. Am J Emerg Med (2021) 41:55–9. doi: 10.1016/j.ajem.2020.12.036 33387929

[31] Gong C Hoff JT Keep RF. Acute inflammatory reaction following experimental intracerebral hemorrhage in rat. Brain Res (2000) 871:57–65. doi: 10.1016/S0006-8993(00)02427-6 10882783

[32] Regolo M Vaccaro M Sorce A Stancanelli B Colaci M Natoli G. Neutrophil-to-Lymphocyte ratio (NLR) is a promising predictor of mortality and admission to intensive care unit of COVID-19 patients. J Clin Med (2022) 11(8):2235. doi: 10.3390/jcm11082235 35456328 PMC9027549

[33] Ben Jemaa A Salhi N Ben Othmen M Ben Ali H Guissouma J Ghadhoune H. Evaluation of individual and combined NLR, LMR and CLR ratio for prognosis disease severity and outcomes in patients with COVID-19. Int Immunopharmacol (2022) 109:108781. doi: 10.1016/j.intimp.2022.108781 35461157 PMC9015974

[34] Zhang WB Tang TC Zhang AK Zhang ZY Hu QS Shen ZP. A clinical prediction model based on post Large artery atherosclerosis infarction pneumonia. Neurologist (2022). doi: 10.1097/NRL.0000000000000434 35353784

[35] Favas TT Dev P Chaurasia RN Chakravarty K Mishra R Joshi D. Neurological manifestations of COVID-19: a systematic review and meta-analysis of proportions. Neurol Sci (2020) 41(12):3437–70. doi: 10.1007/s10072-020-04801-y PMC7577367 33089477

[36] Aksu K Donmez A Keser G. Inflammation-induced thrombosis: Mechanisms, disease associations and management. CurrPharm Des (2012) 18:1478–93. doi: 10.2174/138161212799504731 22364132

[37] Branchford BR Carpenter SL. The role of inflammation in venous thromboembolism. Front Pediatr (2018) 23(6):142. doi: 10.3389/fped.2018.00142 PMC5974100 29876337

[38] Koupenova M Clancy L Corkrey H Freedman J. Circulating platelets as mediators of immunity, inflammation, and thrombosis. Circ Res (2018) 122:337351. doi: 10.1161/CIRCRESAHA.117.310795 PMC5777300 29348254

[39] Krenn-Pilko S Langsenlehner U Thurner EM Stojakovic T Pichler M Gerger A. The elevated preoperative platelet-to-lymphocyte ratio predicts poor prognosis in breast cancer patients. Br J Cancer (2014) 110(10):2524–30. doi: 10.1038/bjc.2014.163 PMC4021515 24675383

[40] Ferroni P Riondino S Formica V Cereda V Tosetto L Farina F. Venousthromboembolism risk prediction in ambulatory cancer patients. Clinical significance of neutrophil/lymphocyte ratio and platelet/lymphocyte ratio. Int J Cancer (2014) 136:1234–40. doi: 10.1002/ijc.29076 25042739

[41] Ozcan Cetin EH Cetin MS Canpolat U Akdi A Aras D Temizhan A. Platelet-to-lymphocyte ratio as a novel marker of in-hospital and long-term adverse outcomes among patients with acute pulmonary embolism: A single center large-scale study. Thromb Res (2017) 150:33–40. doi: 10.1016/j.thromres.2016.12.006 28011405

[42] Tao C Wang J Hu X Ma J Li H You C. Clinical value of neutrophil to lymphocyte and platelet to lymphocyte ratio after aneurysmal subarachnoid hemorrhage. Neurocrit Care (2017) 26:393–401. doi: 10.1007/s12028-016-0332-0 28028791

[43] Zou Y Zhang W Huang C Zhu Y. Clinical significance of neutrophil to lymphocyte ratio and platelet to lymphocyte ratio in acute cerebral hemorrhage with gastrointestinal hemorrhage, and logistic regression analysis of risk factors. Exp Ther Med (2019) 18(3):1533–8. doi: 10.3892/etm.2019.7778 PMC6676203 31410106

[44] Fisher M Meiselman HJ. Hemorheological factors in cerebral ischemia. Stroke (1991) 22(9):1164–9. doi: 10.1161/01.STR.22.9.1164 1833861

[45] Wilhelmsen L Svardsudd K Korsan-Bengtsen K Larsson B Welin L Tibblin G. Fibrinogen as a risk factor for stroke and myocardial infarction. N Engl J Med (1984) 311(8):501–5. doi: 10.1056/NEJM198408233110804 6749207

[46] Woodward M Lowe GD Campbell DJ Colman S Rumley A Chalmers J. Associations of inflammatory and hemostatic variables with the risk of recurrent stroke. Stroke (2005) 36(10):2143–7. doi: 10.1161/01.STR.0000181754.38408.4c 16151030

[47] Di Napoli M Papa F. Inflammation, hemostatic markers,and antithrombotic agents in relation to long-term risk of new cardiovascular events infirst-ever ischemic stroke patients. Stroke (2002) 33(7):1763–71. doi: 10.1161/01.STR.0000019124.54361.08 12105349

[48] Wen H Chen Y. The predictive value of platelet to lymphocyte ratio and d-dimer to fibrinogen ratio combined with WELLS score on lower extremity deep vein thrombosis in young patients with cerebral hemorrhage. Neurol Sci (2021) 42(9):3715–21. doi: 10.1007/s10072-020-05007-y 33443669

