# Supplementary material for: The clinical value of neutrophil-to-lymphocyte ratio, platelet-to-lymphocyte ratio, and D-dimer-to-fibrinogen ratio for predicting pneumonia and poor outcomes in patients with acute intracerebral hemorrhage
Source: Front Immunol. 2022 Oct 10;13:1037255. doi: 10.3389/fimmu.2022.1037255 (PMC9589455; doi:10.3389/fimmu.2022.1037255)
Supplement: Supplementary file 1 [file DataSheet_1.docx]

**Supplementary Materials**

**Supplementary table 1: The detailed clinical characteristics of all patients**

| **Variables** | **All patients (n=329)** | **Statistical method** |
| --- | --- | --- |
| **Demographic** |  |  |
| Age, year, Mean(SD) | 60.97(12.60) | Independent sample t-test |
| Sex, male, n(%) | 210（63.8%） | chi-square test |
| **Clinical characteristics** |  |  |
| Alcohol consumption, n (%) | 27（8.2%） | chi-square test |
| Smoking, n (%) | 40（12.2%） | chi-square test |
| Diabetes mellitus, n (%) | 43（13.1%） | chi-square test |
| History of hypertension, n(%) | 214（65%） | chi-square test |
| Admission SBP, mmHg, Mean(SD) | 169.29(26.84) | Independent sample t-test |
| Admission DBP, mmHg, Mean(SD) | 96.65(16.58) | Independent sample t-test |
| Admission GCS score, median (IQR) | 9.43[5-14] | nonparametric Rank-sum test |
| Platelets,10^9^/l, Mean(SD) | 213.21(74.90) | Independent sample t-test |
| Leukocytes, 10^9^/L, Mean(SD) | 10.43(4.55) | Independent sample t-test |
| Neutrophils, 10^9^/L, Mean(SD) | 8.51(4.46) | Independent sample t-test |
| Lymphocyte , 10^9^/L, Mean(SD) | 1.42(1.05) | Independent sample t-test |
| D-Dimer, ng/mL, Mean(SD) | 766.93(1784.87) | Independent sample t-test |
| Fibrinogen, g/L, Mean(SD) | 2.90(1.14) | Independent sample t-test |
| NLR, Mean(SD) | 9.26(8.98) | Independent sample t-test |
| PLR, Mean(SD) | 209.05(144.88) | Independent sample t-test |
| DFR, Mean(SD) | 301.94(776.29) | Independent sample t-test |
| **Imaging features** |  |  |
| Baseline ICH volume, mL, median (IQR) | 29.61[6.86-43.24] | nonparametric Rank-sum test |
| IVH presence, n (%) | 183（55.6%） | chi-square test |
| SAH presence, n (%) | 69（21%） | chi-square test |
| **ICH Locations** |  |  |
| Basal ganglia hemorrhage, n (%) | 110（33.4%） | chi-square test |
| Thalamic hemorrhage, n (%) | 57(17.3%) | chi-square test |
| Lobar hemorrhage, n (%) | 20(6.1%) | chi-square test |
| Infratentorial hemorrhage, n (%) | 35(21.2%) | chi-square test |
| **Outcome** |  |  |
| In-hospital mortality, n (%) | 18(5.5%) | chi-square test |
| 90-day mortality, n (%) | 87(26.4%) | chi-square test |
| 90-day poor outcome, n (%) | 175(53.2%) | chi-square test |
| 90-day mRS score, median (IQR) | 3.56[2-6] | nonparametric Rank-sum test |

ICH: intracerebral hemorrhage, CT: computed tomography, GCS: Glasgow Coma Scale, IVH: intraventricular hemorrhage, SAH: subarachnoid hemorrhage, INR: interquartile range, SD: standard deviation, mRS: modified Rankin scale, SBP: systolic blood pressure, DBP: diastolic blood pressure, NLR: neutrophil-to-lymphocyte ratio, PLR: platelet-to-lymphocyte ratio, DFR: D-dimer-to-fibrinogen ratio

1. **Statistical method**

All data have passed the normality judgment. When the data conform to the normal distribution, we use the t-test, and indicate the t-value and P-value. We used a nonparametric test when the data did not obey a normal distribution. The non-parametric data should be expressed as medians with interquartile range, with Z-values and P-values marked. For data with dichotomous variable, chi-square test was used, and x^2^-values and P-values were annotated. In this study, the variables using independent sample t-test included age, admission SBP, Admission DBP, platelets, leukocytes, neutrophils, lymphocyte, D-Dimer, fibrinogen, NLR, PLR, and DFR. The variables using chi-square test: sex, alcohol consumption, smoking, diabetes mellitus, history of hypertension, IVH presence, SAH presence, basal ganglia hemorrhage, thalamic hemorrhage, lobar hemorrhage, and infratentorial hemorrhage, in-hospital mortality, 90-day mortality, and 90-day poor outcome.
